# Comment on “Necrotizing Urethritis due to *Aerococcus urinae*”

**DOI:** 10.1155/2015/490560

**Published:** 2015-08-18

**Authors:** Magnus Rasmussen

**Affiliations:** Division of Infection Medicine, Department of Clinical Sciences, Lund University, Tornavägen 10, BMC B14, 221 84 Lund, Sweden

I read with interest the case report of a man with necrotizing urethritis caused by* Aerococcus urinae* by Babaeer and coworkers [1]. This report clearly describes a novel and serious clinical presentation of an infection claimed to be caused by* Aerococcus urinae*. Soft tissue infections in the genital area caused by this organism have been described previously [2, 3] but the case described by Babaeer et al. adds important information on a severe course of infection. However, important questions regarding the aetiology in this case remain unanswered.

Species determination of aerococci is a difficult task with many pitfalls [4]. Therefore, it is vital for the reader to know how the identification of* A. urinae* in the urine culture was made in the present case. Of even greater importance, the reader needs to know if the “*Alpha Haemolytic Streptococcus*” isolated from tissue and from blood also could represent* A. urinae*. Without an explanation of the bacteriological investigations of the isolates from this patient, it is hard to determine the bacterial aetiology of the infection described.
